# Concurrent Renal Cell Carcinoma Diagnosis in a Married Couple: Risk Factors and Unmet Survivorship Needs

**DOI:** 10.7759/cureus.83528

**Published:** 2025-05-05

**Authors:** Matthew Lee, Liwei Jia, Richard Ahn, Jue Wang

**Affiliations:** 1 Medicine, University of Texas Southwestern Medical Center, Dallas, USA; 2 Pathology, University of Texas Southwestern Medical Center, Dallas, USA; 3 Radiology, University of Texas Southwestern Medical Center, Dallas, USA; 4 Internal Medicine: Hematology and Oncology, Harold C. Simmons Comprehensive Cancer Center, University of Texas Southwestern Medical Center, Dallas, USA

**Keywords:** cancer survivorship, caregiver burnout, concurrent diagnosis, financial strain, married couple, psychosocial impact, renal cell carcinoma, survivorship care

## Abstract

Renal cell carcinoma (RCC) is the most common type of kidney cancer, but it is exceedingly rare for both partners in a married couple to be diagnosed with RCC within a short time frame. This case report describes a married couple who were diagnosed with RCC in close succession, exploring potential risk factors and highlighting the unmet survivorship needs of couples affected by cancer. Both partners shared risk factors, including a history of smoking and hypertension. The case focuses on the role of shared environmental exposures, specifically the wife’s occupation as a house cleaner and the husband’s role as a floor installer, in potentially contributing to carcinogenic risks in occupational settings. However, no hereditary RCC syndromes were identified. This report emphasizes the need for integrated care models that address the unique challenges faced by couples with concurrent cancer diagnoses, ensuring comprehensive support for both patients and their caregivers.

## Introduction

Renal cell carcinoma (RCC) is the most common form of kidney cancer, accounting for approximately 80% of all kidney malignancies and 74,000 new cases in the US each year [1,2]. Although most cases of RCC are sporadic, certain genetic predispositions, environmental exposures, and comorbid conditions can contribute to the development of RCC. The concurrent diagnosis of RCC in a married couple in a short timeframe is an exceptionally rare occurrence, raising questions about potential shared risk factors, disease etiology, and the psychosocial impact on affected couples.

Cancer diagnoses in family members, particularly in spouses, can place significant emotional and physical burdens on individuals and have been associated with increased marital distress [3]. While much focus has been placed on the individual experience of cancer, the unique challenges seen in couples diagnosed with concurrent malignancies are underexplored. The strain in marriage, caregiver responsibilities, financial stability, and emotional well-being can be exacerbated when both partners are affected by malignancy.

This case report aims to explore the rare occurrence of concurrent RCC diagnoses in a married couple, investigating potential shared risk factors and highlighting the unmet survivorship needs that arise in such situations.

## Case presentation

**Figure 1:**
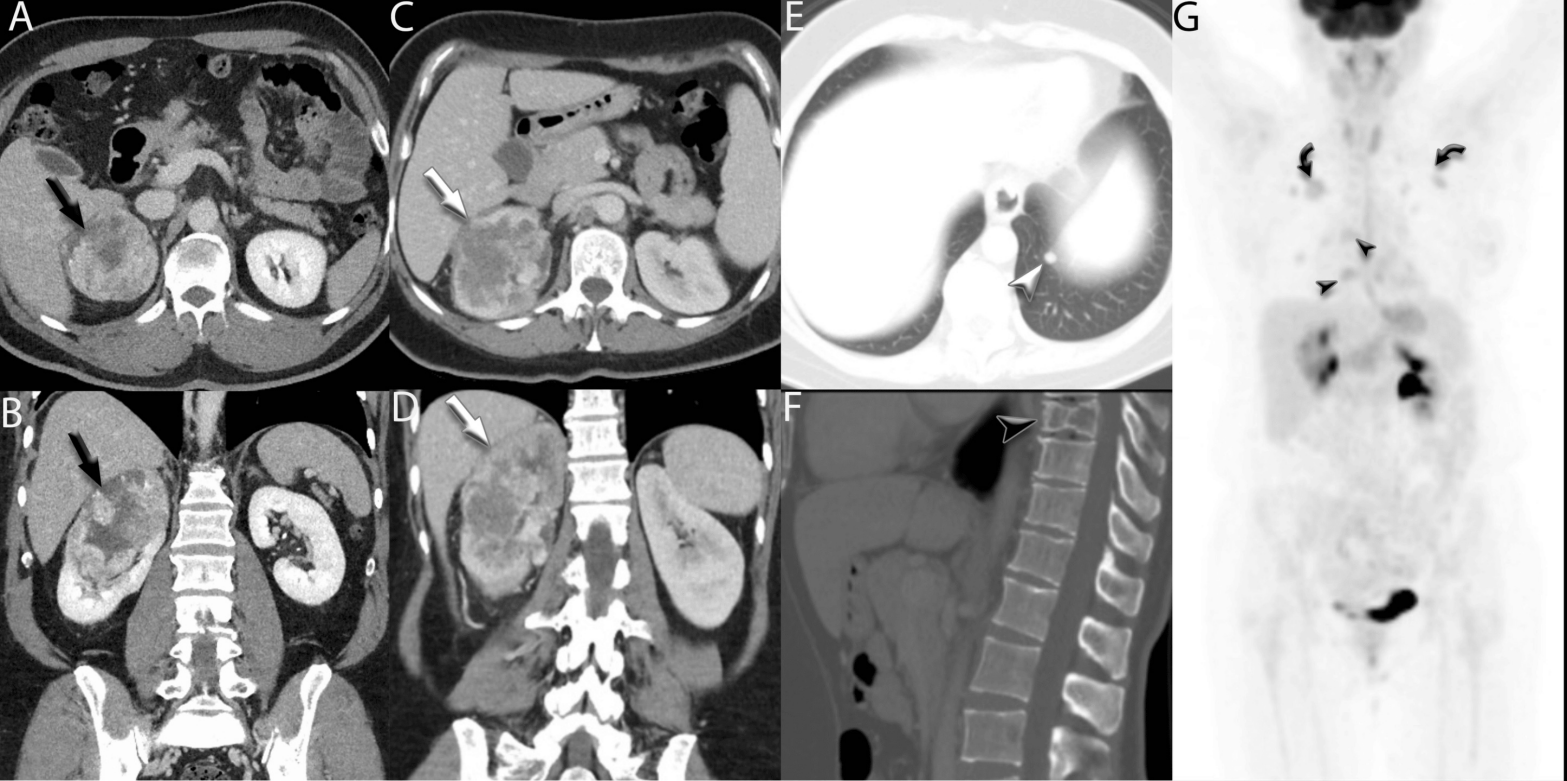
Radiographic findings Axial (A) and coronal (B) CT images demonstrating a large, heterogenous right renal mass (black arrows) in a 52-year-old man. No convincing evidence of metastatic disease was seen on CT of the chest, abdomen, or pelvis. Axial (C) and coronal (D) CT images demonstrating a similar heterogeneous right renal mass in a 47-year-old woman (white arrows). Additional findings suspicious for metastatic disease were seen, including a small left lower lobe pulmonary nodule (E, white arrowhead) and a lytic lesion in the T9 vertebral body (F, black arrowhead). PET/CT MIP image from a follow-up three months after baseline demonstrates numerous FDG-avid lesions in the lungs (curved black arrows) and bones (black arrowheads). PET/CT: positron emission tomography/computed tomography; MIP: maximum intensity projection; FDG: fluorodeoxyglucose

A 47-year-old female, employed as a house cleaner, with a past medical history of hypertension and chronic kidney disease, presented to the hospital with gross hematuria and right flank pain. Initial CT imaging revealed a 7 cm lobulated mass in her right kidney along with bilateral pulmonary nodules and spinal lesions in T7, T9, and L3, concerning for metastatic disease (Figure 1).

**Figure 2:**
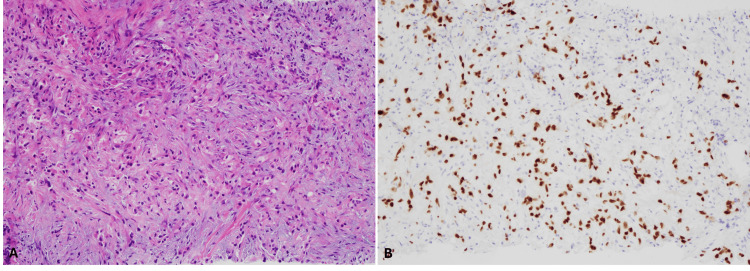
Pathological findings in a 47-year-old female The biopsy of the lung lesion showed infiltrating tumor cells (A), and the renal origin was confirmed by immunohistochemical stain for PAX8 (B).

A subsequent biopsy of the renal mass in March 2021 confirmed clear cell RCC (ccRCC) with sarcomatoid features, a rare and aggressive subtype. To further assess the extent of disease, a biopsy was performed on the lung nodules, which confirmed that her metastatic disease was also ccRCC (Figure 2).

The patient began systemic therapy with pembrolizumab and axitinib in February 2021. She also underwent radiation therapy to her lungs and spine for symptomatic relief. Despite treatment, she developed progressive metastatic disease. Following a lapse in care due to insurance changes, the patient re-established care in July 2022 and was initiated on lenvatinib and everolimus. Over the following year, she underwent radiation to osseous metastatic lesions in her skull, pelvis, and femur. By July 2023, she transitioned to fifth-line therapy with tivozanib and continues to require intensive care due to the aggressive nature of her condition.

**Figure 3:**
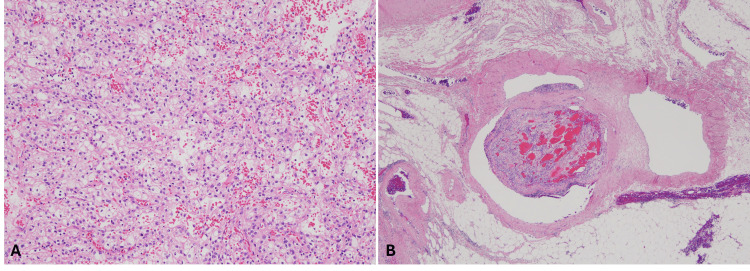
Pathologic findings at the time of nephrectomy for an 8.7 cm renal mass in a 53-year-old man, husband of the previous patient The histological evaluation showed clear cell renal cell carcinoma (RCC) with WHO/ISUP (World Health Organization/International Society of Urological Pathology) grade 3 (A). The tumor extended into the segmental branches of the renal vein (B).

As she battled metastatic disease, her husband, a 53-year-old male, presented to the clinic in November 2022 with a three-month history of unexplained weight loss, fatigue, and intermittent abdominal pain. He had a long-standing history of hypertension and worked as a floor installer. A CT scan revealed a 10.1 cm right renal mass (Figure 1A, 1B). Radical nephrectomy confirmed stage III ccRCC (pT3aN0), with no evidence of lymph node involvement but some perinephric fat invasion (Figure 3).

The husband received one year of adjuvant pembrolizumab without significant side effects. Post-surgical recovery was uneventful, and his disease was monitored with regular imaging and laboratory tests. He remained in remission with no signs of recurrence or metastasis and was under surveillance. While managing his own recovery, he resumed full-time work while caring for his wife during her intensive treatment, compounding the emotional and physical strain of their concurrent diagnoses.

Both patients, although treated for RCC at different stages, faced significant challenges with the diagnosis and the physical, emotional, and psychological demands of cancer treatment. They had difficulty managing the dual burden of illness and caregiving responsibilities, and the lack of coordinated support for couples dealing with cancer became evident throughout the treatment process. Their adult children, aged 24 and 28, assumed primary caregiving roles, which placed significant emotional, physical, and financial strain on them.

## Discussion

This case highlights the rare and complex situation of a married couple who were both diagnosed with RCC within a short time frame. The couple also had very contrasting clinical courses. The wife presented with an aggressive variant of metastatic RCC, and her husband was diagnosed with stage 3 ccRCC within one year. This illustrates the importance of understanding shared and individual risk factors, the psychosocial impact of cancer on couples, and the unmet survivorship needs in such challenging scenarios.

While approximately 90-95% of RCC cases are sporadic, the co-occurrence of RCC in a married couple raises important questions about shared risk factors that may have increased their risk [4]. Several studies have shown that there is a higher incidence of cancer clustering among married couples compared to those who are not couples. A nationwide population-based cohort study in Taiwan found that the risk of cancer clustering among couples was significantly higher than that among those who are not couples, with an incidence of 13.70% in couples versus 11.84% in those who are not couples [5]. Another study conducted in Japan reported that wives whose husbands developed cancer were at an increased risk of developing cancer themselves [6]. Overall, the accumulated evidence indicates that shared environmental exposures, socioeconomic status, lifestyle behaviors, dietary habits, and psychological stress are key factors contributing to the higher incidence of cancer among married couples. However, it's essential to note that these factors may not be deterministic but rather influence the likelihood of cancer development. Further research into these areas is necessary to better understand how shared risk factors among married couples can contribute to cancer incidence. Reporting these cases is crucial for knowledge accumulation in this area, helping guide future research and inform public health strategies. Additionally, this information is vital for health care planning, including cancer survivorship, as it can help tailor more effective prevention and support programs for affected individuals and families.

Interestingly, although smoking is the most recognized risk factor for developing RCC, both our patients were never-smokers [7]. However, both patients had long-standing hypertension, which has been established as a risk factor for RCC in a dose-response relationship [7,8]. This risk factor is particularly important given that both patients had limited access to primary care due to being uninsured, which likely contributed to inadequate hypertension management. Furthermore, both patients are Hispanic Americans, which is an ethnic group found to have nearly three times the odds of developing RCC compared to White Americans [9,10]. Beyond these socioeconomic factors, environmental exposures may have also played a role in the couple’s diagnoses. The husband worked as a floor installer, potentially exposing him to chemicals linked to RCC. For instance, per- and polyfluoroalkyl substances (PFAS) are found in numerous flooring products and have been linked to an increased risk for developing RCC [11]. Also, as a house cleaner, the wife was exposed to numerous cleaning chemicals, such as benzenes, which are a risk factor for RCC [7,12]. Both the husband and wife may have also been exposed to asbestos.

**Table 1:** Comparison of environmental and clinical risk factors, diagnosis, management, and outcomes in a married couple with RCC The wife, exposed to environmental toxins as a house cleaner, presents with advanced metastatic RCC and requires multiple therapies. The husband, a floor installer, diagnosed with stage III RCC, remains in remission with adjuvant pembrolizumab. RCC: renal cell carcinoma

Risk Factor	Wife	Husband
Age (years)	47	53
Gender	Female	Male
Family History of Renal Cell Carcinoma	No	No
Occupation	House cleaner	Floor installer
Environmental Exposure	Cleaning agents and environmental toxins	Asbestos, chemicals, and dust from construction materials
Hypertension	Present	Present
Chronic Kidney Disease	Present	Absent
Renal Mass Size	7 cm	10.1 cm
Metastatic Disease	Present (lung, bone, liver)	Absent
Histological Features	Clear cell RCC with sarcomatoid features	Clear cell RCC
Systemic Therapy	Multiple lines of systemic therapies	Nephrectomy followed by one year of adjuvant pembrolizumab
Radiation Therapy	Received to lungs, spine, and osseous metastatic lesions	None
Outcome	Progressive despite therapy	In remission
Follow-up	Under intensive care and regular follow-up	Regular follow-up with imaging and lab tests

There are studies illustrating an increased risk of developing mesothelioma in family members of workers exposed to asbestos [13]. However, there are mixed results about whether asbestos is a risk factor for RCC [14,15]. There is only one previous case report illustrating a married couple who both developed RCC, and the husband had a long history of asbestos exposure, as he worked as a shipyard worker [16]. Given their occupational risk factors, it is possible that carcinogens from their respective workplaces were transferred through clothing and hair, increasing their risk of developing RCC.

Although emotional strain is common among cancer patients and family members, this couple’s experience was uniquely complex given their simultaneous diagnoses and contrasting disease courses. Among patients with RCC, married patients have better outcomes than widowed and single patients [17]. The reason is likely multifactorial, from stronger financial resources to better support systems. However, this protective effect may not extend to married couples when both partners are simultaneously battling malignancy. The wife experienced persistent anxiety and fear, which worsened as her disease progressed through multiple lines of therapy and when her husband was subsequently diagnosed with RCC. In contrast, the husband’s disease was surgically treated with a favorable prognosis. This dynamic caused the husband to struggle with feelings of guilt and self-blame. Although feelings of guilt and self-criticism are common in cancer patients, his feelings of guilt were intensified, as he felt his occupational exposures may have contributed to both of their illnesses [18]. Their experience highlights the unique emotional toll that emerges when married partners both face cancer diagnoses.

**Table 2:** Summary of unmet survivorship needs in a married couple with RCC and their care providers The wife faces significant challenges due to disease progression, while the husband, in remission, bears the emotional and physical strain of caregiving. Their children, aged 24 and 28, experience considerable stress as primary caregivers, highlighting the need for comprehensive support systems for both patients and their families. RCC: renal cell carcinoma

Unmet Survivorship Need	Wife	Husband	Daughter	Son
Psychosocial Support	Emotional strain from aggressive disease	Emotional burden from caregiving and health recovery	Emotional distress from witnessing illness	Emotional strain from balancing caregiving and personal life
Financial Assistance	Financial burden from treatment and insurance lapses	Limited by caregiving and health-related expenses	Potential financial strain from caregiving duties	Potential financial strain from caregiving duties
Palliative Care	Ongoing need for palliative care	No	Needs guidance on supporting the mother’s decline	Needs support in managing both parents' health
Treatment Access and Adherence	Interrupted care due to insurance issues	No interruptions, adheres to follow-up care	Managing mother’s treatment adherence	Helps with scheduling and follow-up care
Physical Rehabilitation	Yes	Yes	Assists with rehabilitation exercises	Helps with mobility and rehab tasks
Caregiver Support	Lack of formal caregiver support	Burden of caregiving while managing personal health	Significant caregiving responsibilities	Shares caregiving duties, potential stress
Support for Work and Lifestyle	Unable to work	Resumed full-time work despite caregiving strain	Need to adjust work hours for caregiving	Balances work and caregiving duties
Communication With the Care Team	Inconsistent communication during treatment gaps due to illness	Regular communication with the care team	Needs clear information on treatment plans	Needs help coordinating care plans
Long-Term Monitoring and Follow-Up	Needs extensive follow-up	Routine follow-up for remission surveillance	Needs to help manage health monitoring	Needs assistance with follow-up coordination
Health-Related Quality of Life	Decreased due to disease progression	Impacted by caregiving stress	Affected by caregiving stress	Impacted by caregiving responsibilities

This case also highlights significant challenges in survivorship care for families navigating concurrent cancer diagnoses. While recovering from his nephrectomy, the husband returned to full-time work and was also managing the care of his wife. However, coordinating follow-up appointments proved difficult given the husband’s full-time work schedule, his own medical treatments, and his wife’s ongoing treatments. Although the wife utilized individual counseling services, there were no integrated counseling programs designed to support both partners through their concurrent diagnoses. Family and couples therapy can reduce distress and improve coping for both cancer patients and caregivers, and the need for joint therapy may be even greater when the caregiver is also managing their own concurrent cancer diagnosis [19]. The couple also faced significant financial strain due to their inability to work during their treatments. The wife experienced a lapse in insurance during a critical time of disease progression, but she was able to receive care after being approved for a financial assistance program at a safety-net hospital. Financial counseling and assistance programs are essential for families navigating concurrent cancer diagnoses. As seen by their challenges, a coordinated survivorship plan that incorporates joint counseling, same-day follow-up care, practical caregiver support strategies, and financial counseling is essential for couples dealing with concurrent cancer diagnoses.

## Conclusions

In conclusion, this case report highlights the diagnosis of renal cell carcinoma (RCC) in a married couple within a span of one year, shedding light on the potential role of shared environmental exposures, lifestyle behaviors, and other contributing factors in the development of cancer among married couples. While these factors are not deterministic, they likely influence the increased likelihood of cancer in this case. The report emphasizes the importance of documenting such cases to enrich our understanding of cancer incidence in couples and contributes to the accumulation of knowledge in this area. Moreover, the findings underscore the significance of considering these shared risk factors in health care planning, especially in cancer survivorship programs, to better support individuals and families affected by RCC and other cancers. Further research is necessary to explore the intricate relationship between these factors and cancer development, paving the way for more effective prevention and treatment strategies.

## References

[1] Escudier B, Porta C, Schmidinger M (2019). Renal cell carcinoma: ESMO Clinical Practice Guidelines for diagnosis, treatment and follow-up. Ann Oncol.

[2] Tran J, Ornstein MC (2022). Clinical review on the management of metastatic renal cell carcinoma. JCO Oncol Pract.

[3] Wang Y, Feng W (2022). Cancer-related psychosocial challenges. Gen Psychiatr.

[4] Alchoueiry M, Cornejo K, Henske EP (2024). Kidney cancer: links between hereditary syndromes and sporadic tumorigenesis. Semin Diagn Pathol.

[5] Wang JY, Liang YW, Yeh CC, Liu CS, Wang CY (2018). Time-dependent risks of cancer clustering among couples: a nationwide population-based cohort study in Taiwan. BMJ Open.

[6] Izumi S, Imai K, Nakachi K (2004). Excess concordance of cancer incidence and lifestyles in married couples (Japan): survival analysis of paired rate data. Cancer Causes Control.

[7] Navai N, Wood CG (2012). Environmental and modifiable risk factors in renal cell carcinoma. Urol Oncol.

[8] Chow WH, Gridley G, Fraumeni JF Jr, Järvholm B (2000). Obesity, hypertension, and the risk of kidney cancer in men. N Engl J Med.

[9] Padala SA, Barsouk A, Thandra KC (2020). Epidemiology of renal cell carcinoma. World J Oncol.

[10] Batai K, Harb-De la Rosa A, Lwin A, Chaus F, Gachupin FC, Price E, Lee BR (2019). Racial and ethnic disparities in renal cell carcinoma: an analysis of clinical characteristics. Clin Genitourin Cancer.

[11] Rhee J, Chang VC, Cheng I (2023). Serum concentrations of per- and polyfluoroalkyl substances and risk of renal cell carcinoma in the multiethnic cohort study. Environ Int.

[12] Pesch B, Haerting J, Ranft U, Klimpel A, Oelschlägel B, Schill W (2000). Occupational risk factors for renal cell carcinoma: agent-specific results from a case-control study in Germany. Int J Epidemiol.

[13] Ferrante D, Bertolotti M, Todesco A, Mirabelli D, Terracini B, Magnani C (2007). Cancer mortality and incidence of mesothelioma in a cohort of wives of asbestos workers in Casale Monferrato, Italy. Environ Health Perspect.

[14] Peters CE, Parent MÉ, Harris SA, Kachuri L, Latifovic L, Bogaert L, Villeneuve PJ (2018). Workplace exposure to asbestos and the risk of kidney cancer in Canadian men. Can J Public Health.

[15] Pang CC, Phan K, Karim MN, Afroz A, Winter M, Glass DC (2021). Occupational asbestos exposure and kidney cancer: systematic review and meta-analysis of cohort studies. Ann Work Expo Health.

[16] Matsumoto R, Shinohara N, C-Hatanaka K (2015). Concurrent occurrence of renal cell carcinoma with rhabdoid features in a married couple: a case report. BMC Res Notes.

[17] Li Y, Zhu MX, Qi SH (2018). Marital status and survival in patients with renal cell carcinoma. Medicine (Baltimore).

[18] Austin J, Drossaert CH, Sanderman R, Schroevers MJ, Bohlmeijer ET (2021). Experiences of self-criticism and self-compassion in people diagnosed with cancer: a multimethod qualitative study. Front Psychol.

[19] Riba MB, Donovan KA, Andersen B (2019). Distress management, Version 3.2019, NCCN Clinical Practice Guidelines in Oncology. J Natl Compr Canc Netw.

